# Bird preferences for fruit size, but not color, vary in accordance with fruit traits along a tropical elevational gradient

**DOI:** 10.1002/ece3.9835

**Published:** 2023-02-14

**Authors:** Richard J. Hazell, Katerina Sam, Rachakonda Sreekar, Samson Yama, Wulan Koagouw, Alan J. A. Stewart, Mika R. Peck

**Affiliations:** ^1^ Department of Evolution, Behaviour and Environment, School of Life Sciences University of Sussex Brighton UK; ^2^ Biology Centre of Czech Academy of Sciences Institute of Entomology Ceske Budejovice Czech Republic; ^3^ Faculty of Science University of South Bohemia Ceske Budejovice Czech Republic; ^4^ New Guinea Binatang Research Centre Madang Papua New Guinea; ^5^ National Research and Innovation Agency Central Jakarta Indonesia

**Keywords:** artificial fruits, community level, elevation, frugivory, gape width, Papua New Guinea

## Abstract

Birds constitute one of the most important seed dispersal agents globally, especially in the tropics. The feeding preferences of frugivorous birds are, therefore, potentially of great ecological importance. A number of laboratory‐based and observational studies have attempted to ascertain the preferences of certain bird species for certain fruit traits. However, little attention has been paid to community‐wide preferences of frugivorous birds and the impact this may have on fruit traits on a broader scale. Here, we used artificial fruits of different colors and sizes to investigate community‐wide fruit trait preferences of birds at three sites along an elevational gradient in Papua New Guinea. We recorded attack rates on artificial fruits as visible impressions made by a bird's beak during a feeding attempt. We also measured the colors and sizes of real fruits at each site, and the gape widths of frugivorous birds, allowing for comparisons between bird feeding preferences and bird and fruit traits. Regardless of elevation, red and purple fruits were universally preferred to green and attacked at similar rates to one another, despite strong elevational patterns in real fruit color. However, elevation had a significant effect on fruit size preferences. A weak, non‐significant preference for large fruits was recorded at 700 m, while medium fruits were strongly preferred at 1700 m and small fruits at 2700 m. These patterns mirror those of both real fruit size and frugivorous bird gape width along the gradient, suggesting the potential for selective pressure of birds on fruit size at different elevations.

## Graphical abstract

We used artificial fruits to examine avian community‐wide feeding preferences for fruit size and colour on an elevational gradient in Papua New Guinea. We found that birds preferentially selected artificial fruits corresponding to both frugivore gape width and the size of real fruits at each elevation, indicating the potential for selective pressure of birds on fruit size. However, colour preferences of birds did not correspond to real fruit colour prevalence at different elevations.

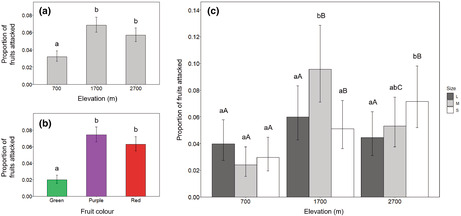

## 1 INTRODUCTION

Seed dispersal is a key factor determining tree community assembly (Harrison et al., 2013; Levin et al., 2003; McConkey et al., 2012). It is estimated that 70%–90% of tropical tree species bear fleshy fruits that are primarily dispersed by vertebrate frugivores (Muller‐Landau & Hardesty, 2005), and that birds represent the majority of these frugivores in most regions (Corlett, 2017; Willson et al., 1989). Feeding preferences of birds thus have the potential to be a significant selective pressure on the evolution of fruit traits (Eriksson, 2016; Lord, 2004), as seed dispersal is known to be related to plant fitness (Beckman & Rogers, 2013; Howe & Smallwood, 1982; Snell et al., 2019). However, the preferences of birds for different fruit traits in different environments are poorly known.

Birds are known to select fruits visually, primarily using cues such as color and size (Corlett, 2011; Duan et al., 2014; Schaefer & Ruxton, 2011). According to zoological classifications of fruit syndromes, bird‐dispersed fruits are typically categorized as brightly colored (Gautier‐Hion et al., 1985; Janson, 1983; Lomáscolo et al., 2008). However, it is not fully understood why birds choose certain fruit colors over others. Some evidence points to fruit color cues signaling high nutritional reward (Cazetta et al., 2012; Schaefer et al., 2008, 2014). Alternatively, an important factor may simply be conspicuousness, i.e., fruits that contrast against a background of foliage are more likely to be noticed by birds (Nevo et al., 2018; Ordano et al., 2017; Schmidt et al., 2004). Direct selection for specific colors based on innate preferences of birds has found little support (Willson et al., 1990).

Fruit size preferences of birds may similarly represent a combination of a choice and physical limitations. Unlike mammalian frugivores, birds usually swallow fruits whole (Wheelwright, 1985), meaning their gape size limits the maximal diameter of fruits they can consume (Corlett, 1998, 2017; Wheelwright, 1985). This imposes an upper limit on the size of seed that a given bird can disperse, although not a lower limit (Wheelwright, 1985). Nevertheless, there is some evidence suggesting that larger birds tend to preferentially feed on larger fruits (Burns, 2013; Chen & Moles, 2015; Sobral et al., 2010a, 2010b). The matching of traits in this way (e.g., fruit size and bird body/gape size) has recently gained attention for its apparent importance in structuring species interaction networks, particularly mutualistic ones such as frugivory (Bender et al., 2018; Dehling, Töpfer, et al., 2014; Garibaldi et al., 2015; González‐Castro et al., 2015; Muñoz et al., 2017).

Determining the relative importance of fruit traits in bird attraction is a major challenge due to the covariation of traits in uncontrollable ways (Levey & Grajal, 1991). The use of artificial fruits is one way to independently manipulate fruit traits. Experiments using artificial fruits have been largely limited to laboratory analyses in which birds feed on gelatin‐ or dough‐based fruits under artificial conditions (Duan et al., 2014; Levey & Grajal, 1991; Puckey et al., 1996; Sallabanks, 1993; Willson et al., 1990). However, it is known that birds under laboratory conditions may exhibit unnatural feeding behaviors (Alves‐Costa & Lopes, 2001). Additionally, the existing studies were generally limited to a few individuals of one to four focal bird species, which limits their broader applicability. If we are to understand the evolutionary implications of bird feeding preferences on a community level, experiments need to be conducted at the community scale.

Field‐based approaches using artificial fruits constructed from waterproof modeling clay offer a solution to this problem. Birds readily attack these fruits but rarely swallow them (Alves‐Costa & Lopes, 2001). Fruits may thus be deployed in the field for a number of days and exposed to the entire frugivore community. Furthermore, the marks left on the fruits reveal some information about the feeding behavior of the birds that attempted to eat them. The fruits are easy to produce in large numbers and traits such as size and color can be precisely and individually manipulated. Few studies have used artificial modeling clay fruits in this way (Alves‐Costa & Lopes, 2001; Cazetta et al., 2012; Ferger et al., 2016; Galetti et al., 2003), and to our knowledge, none have used them to experimentally test avian frugivore preferences of fruit traits (especially size) across environmental gradients.

Tropical mountains provide an opportunity to examine bird preferences for fruit traits in different natural environments. Characterized as they are by rapid changes in environmental conditions across relatively small distances, tropical mountains allow the study of changes in functional trait diversity across habitats without the need for costly regional‐scale survey regimes (Swenson et al., 2011). Plant and bird communities are indeed known to change with elevation, as are their morphological traits (Dehling, Fritz, et al., 2014; Swenson et al., 2011). For example, the mean body size and abundance of avian frugivores are known to reduce with increasing elevation (Sam et al., 2017; Terborgh, 1977), while fruit color and mean fruit size similarly show changing patterns with elevation (Guo et al., 2013; Lu et al., 2019; Zoratti et al., 2015).

Parallel patterns in plant and bird functional trait diversity across elevation should enable the study of inter‐trophic functional relationships such as frugivory, with clear implications for seed dispersal (Dehling, Töpfer, et al., 2014; Onstein et al., 2017). For example, the notion of cause and effect (i.e., the relative importance of frugivores selecting for fruit traits vs. fruit availability determining frugivore community traits) is significant with regard to the evolutionary mechanisms behind seed dispersal and our understanding of the importance of keystone frugivore species (Albert et al., 2020). It is therefore puzzling that the preferences of fruit traits by frugivores have rarely been studied in an elevational context. An experimental approach should help us to determine whether selective pressure by avian frugivory has the potential to account for any observed elevational patterns in fruit traits, by showing us whether frugivore preferences reflect (and thus could potentially have contributed to) existing fruit trait patterns.

Here, we attempt to determine avian frugivore preferences for fruit color and size at different elevations (low, mid, and high: 700, 1700, and 2700 m above sea level, respectively) along a tropical mountain range in Papua New Guinea. This is made possible by determining the number of feeding attempts on artificial modeling clay fruits of different colors (green, purple, and red) and sizes (small, medium, and large: 7, 13, and 19 mm in diameter, respectively). We compare this to the relative color and size prevalence of real fruits, and the gape width of frugivorous birds present at each site. We predict that: (a) birds will prefer fruit colors that are naturally common at a given elevation; (b) birds will prefer fruit sizes that are naturally common at a given elevation, and preferences will reflect bird gape limitation; (c) the number of feeding attempts on artificial fruits will decrease with increasing elevation because a relatively higher abundance of frugivores, which we expect in lowlands, should naturally lead to higher rates of frugivory (Smith & McWilliams, 2014). A negative result for (a) and (b) would indicate that frugivores are unlikely to be having a selective influence on the fruit traits in question. Conversely, while a positive result does not in itself prove a causative effect of frugivory on fruit traits, it would constitute the first evidence of frugivores' fruit trait preferences aligning with existing trait profiles in a natural system.

## 2 MATERIALS AND METHODS

### 2.1 Study sites

**FIGURE A1:**
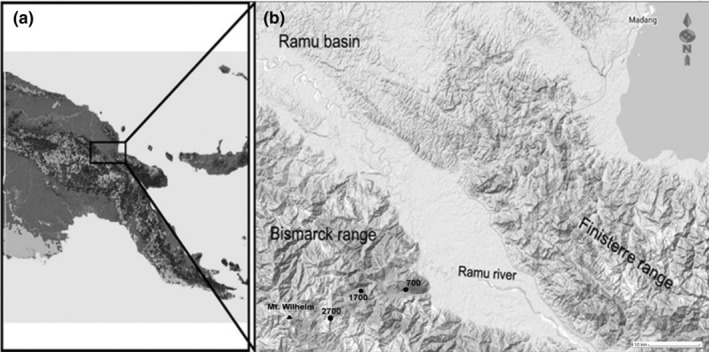
Location of the elevational gradient of Mt. Wilhelm in Papua New Guinea (a) and the study sites along the gradient (b).

We conducted this study along the northeastern slopes of Mt Wilhelm (4509 m) in the northern watershed of the Central Range of Papua New Guinea (Figure A1). The study area is located in the Usino‐Bundi district of southern Madang province and comprises three study sites separated by 1000 m elevation, ranging from 700 to 2700 m above sea level (a.s.l.) (5°43'36" S, 145°15'30" E; 5°48'54" S, 145°09'18" E). The sites represent part of a complete rainforest transect spanning from the lowland floodplains of the Ramu River to the treeline (Sam & Koane, 2014). The habitats at the surveyed sites range from foothill forest (700 m a.s.l.) to lower‐montane forest (1700 m a.s.l.) and mid‐montane forest (2700 m a.s.l.) (Paijmans, 1976). Mean annual temperature recorded using data loggers decreases from 21.97°C at 700 m to 14.34°C at 2700 m. Average annual precipitation measured by local weather stations is 3288 mm in the lowlands, rising to 4400 mm at 2700 m a.s.l., with a distinct condensation zone around 2500–2700 m a.s.l. (Marki et al., 2016; Sam et al., 2017, 2019; Sam & Koane, 2014).

### 2.2 Fruit surveys

Data on size and color of real fruits at each elevation were collected using transects surveys of fruiting woody plants. We created 10 transects at every elevational study site, each measuring 20*500 m, to give a total of 10 ha surveyed per elevation. We collected fruits (both from branches and fallen onto the ground) from all fruiting trees present within the transects. We identified fruiting plants to species level where possible. Collected fruits were measured along their secondary axis, giving a measure of mean fruit diameter per individual plant. The secondary axis was used because this represents the minimal dimensions restricting possible dispersal by gape‐limited frugivores such as birds (Mazer & Wheelwright, 1993). Each collected fruit was photographed and its color was noted. For bicolored fruits, both colors were noted, although only the most dominant color (covering >50% of the fruit's surface) was considered for analysis.

### 2.3 Bird surveys

Bird abundance data were collected using point counts. At each of the three elevations, we surveyed a 2250 m transect comprising 16 points separated by 150 m. Transects predominantly followed those of Sam and Koane (2014). Surveys began at sunrise (approximately 0530 h) and were completed by 1100 h. We replicated the survey methodology three times on 3 different days. Individual point counts lasted 15 min and commenced a few min after arriving at a point to minimize the effects of disturbance caused by arrival (Bibby et al., 2000). We recorded all birds seen or heard within a radius of 50 m. To minimize multiple counts of one individual, we followed the protocol of Sam and Koane (2014): i.e., we only counted multiple conspecifics if two or more individuals could be heard singing simultaneously or from clearly different locations within a period of a few seconds. Points were located using Garmin GPSmap 62S handheld GPS units.

For the analytical purposes of this study, we considered only species richness and abundance of obligate frugivores that are known to feed primarily in the forest understory. Obligate frugivores are known to form a disproportionately important component of plant–frugivore networks in tropical forests (de Assis Bomfim et al., 2018; Palacio et al., 2016). First, birds were classified into feeding guilds based on data from Sam et al. (2017), who analyzed the diets of Mt Wilhelm bird species by using emetic tartar to induce regurgitation. Birds were classified as understory frugivores based on information on foraging strata from Pratt and Beehler (2015) and our own observations. Gape width measurements were taken from museum specimens of birds collected from the Mt Wilhelm study sites and stored at the Natural History Museum of Denmark, University of Copenhagen.

### 2.4 Artificial fruit exposures

**FIGURE 1:**
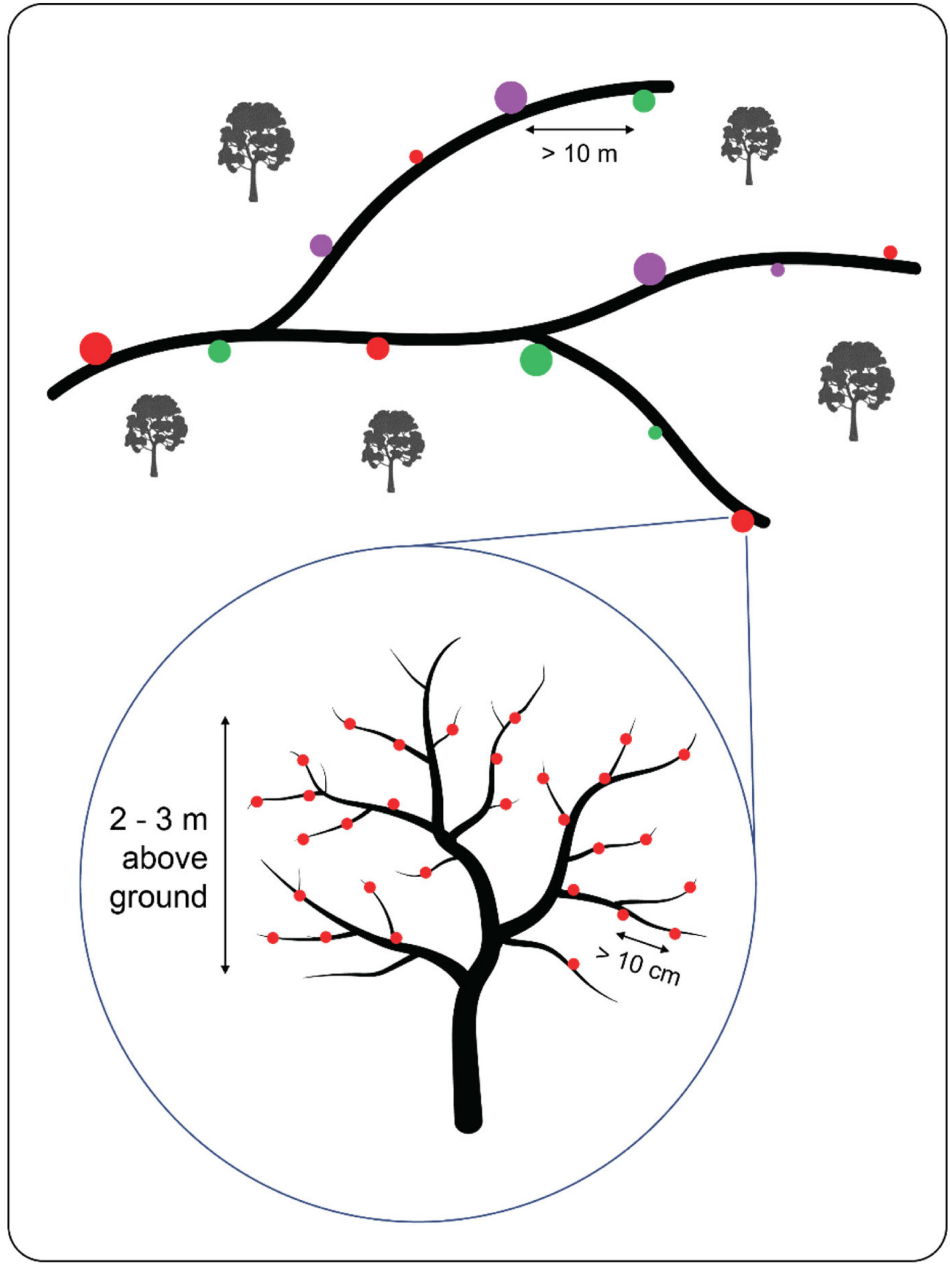
Sketch showing a section of the experimental study design within each elevational site. Black lines indicate forest trails along which clusters of artificial fruits, represented here by colored circles, were exposed on suitable host trees. Each cluster contained 30 fruits of a given color (green, purple, or red) and size (“large,” “medium,” and “small”—19, 13, and 7 mm in diameter, respectively). Six clusters of each unique color/size combination were exposed, giving a total of 54 clusters, at each elevation. The inset shows a close‐up view of an individual fruit cluster (in this case, red/medium size) exposed on a host tree.

Spherical artificial fruits (hereafter “fruits”) were prepared from non‐toxic modeling clay (Koh‐I‐Noor Hardtmuth, Ceske Budejovice, and Czech Republic) (Sam et al., 2015) in three different colors (green, purple, and red) and three different sizes (19, 13, and 7 mm diameter—hereafter “large,” “medium,” and “small,” respectively), giving a total of nine unique size/color combinations. Colors and sizes were selected based on the observed prevalent characteristics of ripe fruits at each of the three survey sites. At each site, 180 artificial fruits of each color/size combination were exposed in six clusters of 30 fruits. This gave a total of 1620 exposed fruits at each elevational study site (30 fruits*6 clusters*3 colors*3 sizes). Each fruit cluster comprised fruits of a single color/size combination (e.g., large red) and was exposed to a separate individual host tree (Figure 1). Host trees were selected such that fruit clusters were located at least 10 m apart from each other, with fruits placed between 2 and 3 m above the ground. Fruits were attached to the host tree using the florist's wire. The fruits were no closer than 10 cm to each other, and no further than 1 m from the end of the branch (Ferger et al., 2016). Host trees were selected based on two criteria: having enough branches to allow the attachment of 30 fruits within the required height, and not currently displaying any fruits of their own or showing evidence of recent fruiting (e.g., decaying fruits on the ground).

Fruits were checked for evidence of potential attack 72 h after initial exposure. At this point, any damaged or removed fruits were replaced. After a further 72 h, the fruits were again checked and removed, giving a total of 144 h (6 days) of exposure time. During both the intermediate check and the final collection, any bitten, pecked, or removed fruits were noted. The taxonomic identity of the attacker was ascertained based on characteristics of the impressions left in the modeling clay (Alves‐Costa & Lopes, 2001). These were categorized broadly as bird, arboreal mammal, bat, and arthropod. For bird‐attacked fruits, additional information was collected on the feeding technique attempted by the attacker based on physical characteristics of the impressions left on the fruit (see below for details).

**FIGURE A2:**
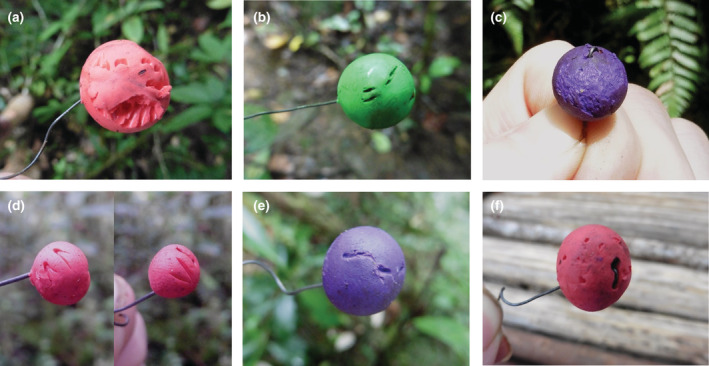
Photographs of artificial fruits showing evidence of attack by different taxa: arboreal mammals (a), bats (b), arthropods (c), and birds (d–f). Bird‐attacked fruits are subdivided into: (d) “held” fruits that had been grasped on both sides (both sides shown here), (e) “intermediate attack” fruits showing imprints from a single feeding attempt whose maximum distance was less than the fruit's diameter, and (f) “pecked” fruits showing small individual holes characteristic of pecking.

We assigned categories to all bird‐attacked fruits (Figure A2): (a) held: these fruits had clearly been grasped in the bill leaving impressions of the mandibles on opposite sides, suggesting the attacker was capable of swallowing the fruit; (b) intermediate: these fruits showed imprints of upper and lower mandibles but whose maximal distance apart was less than the fruit's diameter; an (c) pecked: these fruits showed only a single hole, characteristic of pecking.

All protocols and procedures employed in this study were reviewed and approved by the University of Sussex Sciences & Technology Cross‐Schools Research Ethics Committee.

### 2.5 Data analysis

#### 2.5.1 Real fruit and bird analysis

In order to allow a comparison of bird fruit preferences with the actual existing fruit characteristics and bird gape widths present at each elevation, data analysis was performed on fruit and bird characteristics. Data analysis was carried out as follows:

1. To determine the abundances of real fruit colors corresponding to the fruits represented in this study—green, purple, and red—across elevations (addressing prediction a), we used three separate generalized linear models (GLMs), each with binomial error distributions. The models were named GLM_green fruits_, GLM_purple fruits_, and GLM_red fruits_, and the three response variables were the proportion of all individual fruiting plants that bore green, purple, and red fruits, respectively.
2. To determine the variation in fruit size across elevations (addressing prediction b), we used a GLM with Gaussian error distributions; here, mean fruit diameter per fruiting plant species was our response variable. We termed this model GLM_species fruit diameter_. To characterize the effects of elevation on the upper limit of fruit diameter, we used a linear quantile regression using the *quantreg* package in R (Koenker et al., 2019). We selected the 95th quantile to approximate the upper limit of fruit diameter. In addition, we compared community‐weighted mean fruit diameter across elevations using a separate GLM with Gaussian error distributions, using data weighted by abundance of individual fruiting plants. We termed this model GLM_community fruit diameter_. In this case, the response variable was individual fruit diameter (i.e., species mean fruit diameter weighted by that species' relative abundance).
3. To determine the differences in frugivore gape width across elevations (addressing prediction b), we used a GLM with Gaussian error distributions; here, gape width of each recorded species was our response variable. This model was named GLM_species gape width_. We again used quantile regression (selecting the 95th quantile) to approximate the upper limit of gape width. As with fruit diameter, we performed a separate GLM with Gaussian error distributions using abundance‐weighted data in order to compare community‐weighted mean gape width across elevations. This we termed GLM_community gape width_.
4. To determine the differences in frugivore abundances across elevations (relating to prediction c), we used a GLM with Poisson error distributions; frugivore abundance at each survey point was our response variable. GLM_frugivore abundance_ was the name given to this model.

#### 2.5.2 Artificial fruit exposures

To test whether bird preferences for fruit color and size would differ depending on elevation (addressing predictions a and b, respectively), we modeled the proportion of attacked artificial fruits per 30‐fruit cluster as a function of fruit color, fruit size, elevation, and their interactions using a GLM with binomial error structure and a logit link, with individual fruits acting as the experimental unit. We named this model GLM_attack rates_. Backward elimination procedure was then used to sequentially simplify the model for each variable that was not significant. The importance of the eliminated variable was determined using likelihood ratio tests. Parameters of the final model were considered significant at *p* < .05, and Tukey pairwise comparisons (“emmeans” function in *emmeans* package) (Lenth et al., 2018) were used to adjust *p*‐values during multiple comparisons. Finally, to test for correlations among fruit color, fruit size, and elevation, we calculated scaled generalized variance inflation factors (GVIF) for each independent variable (“vif” function in car package) (Fox & Weisberg, 2019).

In order to test the effect of elevation on the proportion of fruits held in the beak by birds and thus their potential seed dispersal success (relating to prediction b), we used a GLM with binomial error structure to investigate the effect of fruit color, fruit size, elevation, and their interactions on the proportion of artificial fruits per 30‐fruit cluster that had been “held” only (as defined above) while excluding those not held (i.e., “pecked” + “intermediate attack”). We named this model GLM_holding rates_. As above, backward elimination and likelihood ratio tests were used to select an appropriate model, and Tukey pairwise comparisons were used to adjust p‐values during multiple comparisons. We used “held” as a response variable in our model.

## 3 RESULTS

### 3.1 Real fruit and bird data

#### 3.1.1 Fruit color

**FIGURE 2:**
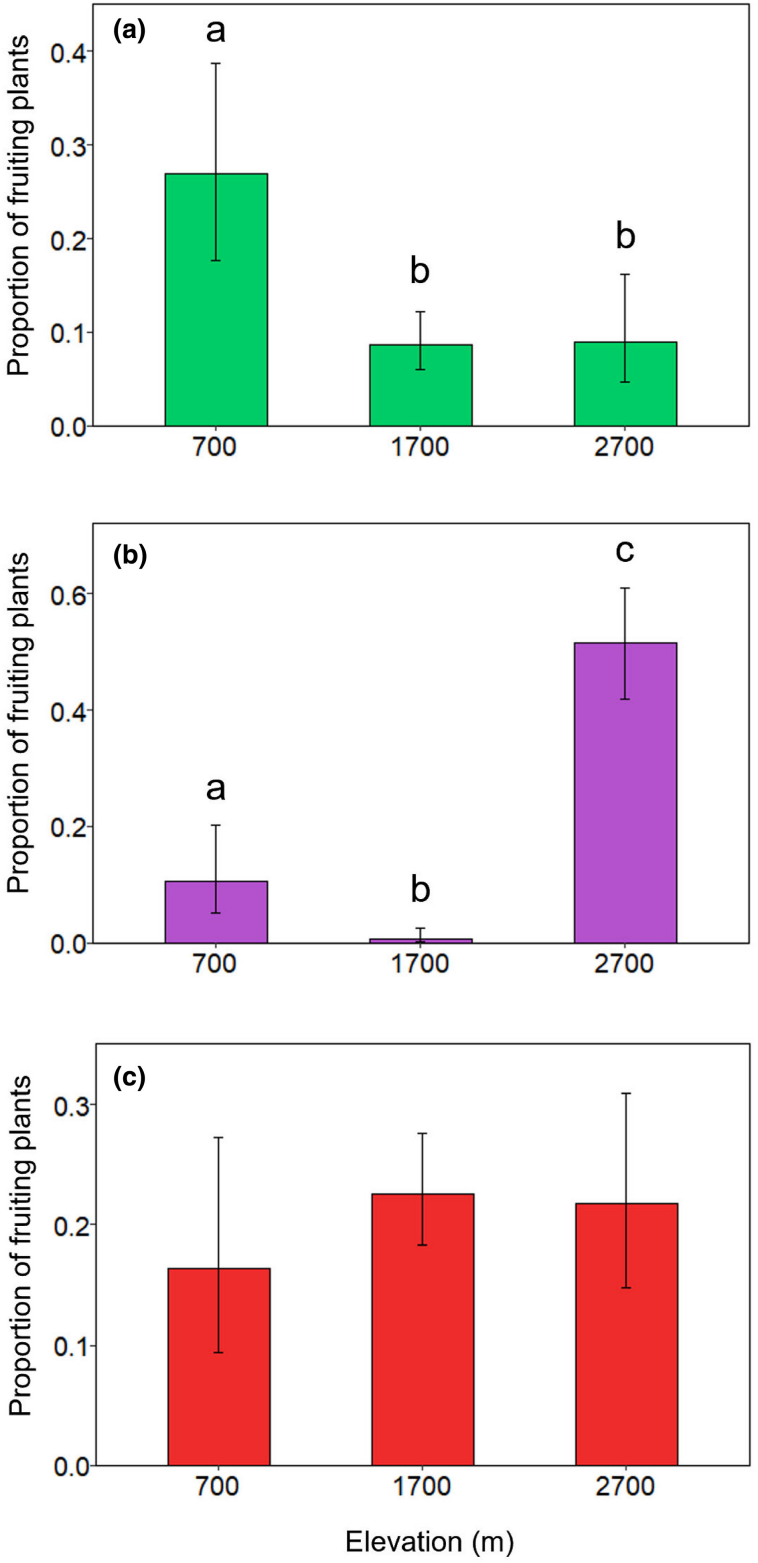
Relative abundance of fruiting plants at each elevation for the fruit colors represented in this study, represented as a proportion of the total number of fruiting trees at each elevation. Mean proportions are displayed for green (a), purple (b), and red (c) fruits. Error bars represent 95% confidence intervals. Letters above bars denote significant differences between elevations (*p* < .05), after adjusting using Tukey pairwise comparisons.

**FIGURE A3:**
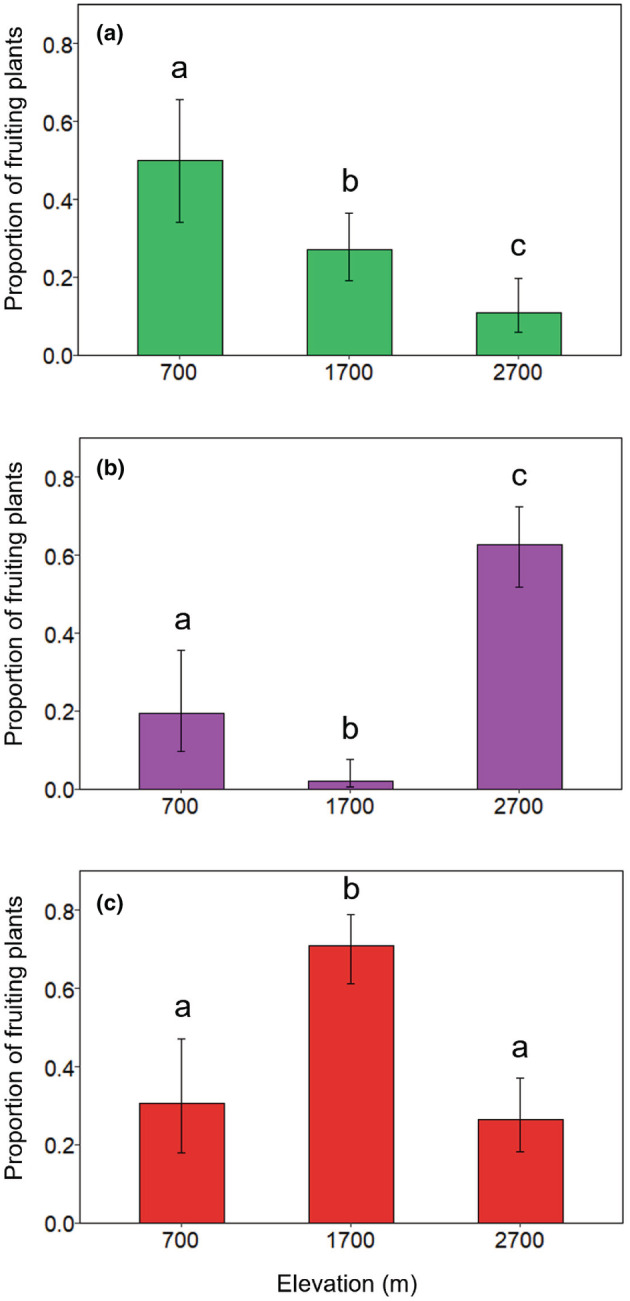
Relative abundance of real fruiting plants at each elevation for the fruit colors represented in this study, represented here as a proportion of the combined total number of plants bearing fruits of those three colors within a given elevation. Mean proportions are displayed for green (a), purple (b), and red (c) fruits. Error bars represent 95% confidence intervals. Letters above columns denote statistically significant differences between elevations (*p* < .05).

The relative abundances of plants naturally bearing green and purple fruits changed significantly with elevation (GLM_green fruits_ and GLM_purple fruits_, respectively, *p* < .01), although this was not the case for plants bearing red fruits (GLM_red fruits_, *p* = .52; Table A1). Plants bearing green fruits were most common at 700 m a.s.l. (Figure 2a), while those bearing purple fruits were most common at 2700 m (Figure 2b). At 1700 m, plants bearing red fruits showed the highest abundance of the three fruit colors included in this study (Figure A3c).

#### 3.1.2 Fruit size

**FIGURE 3:**
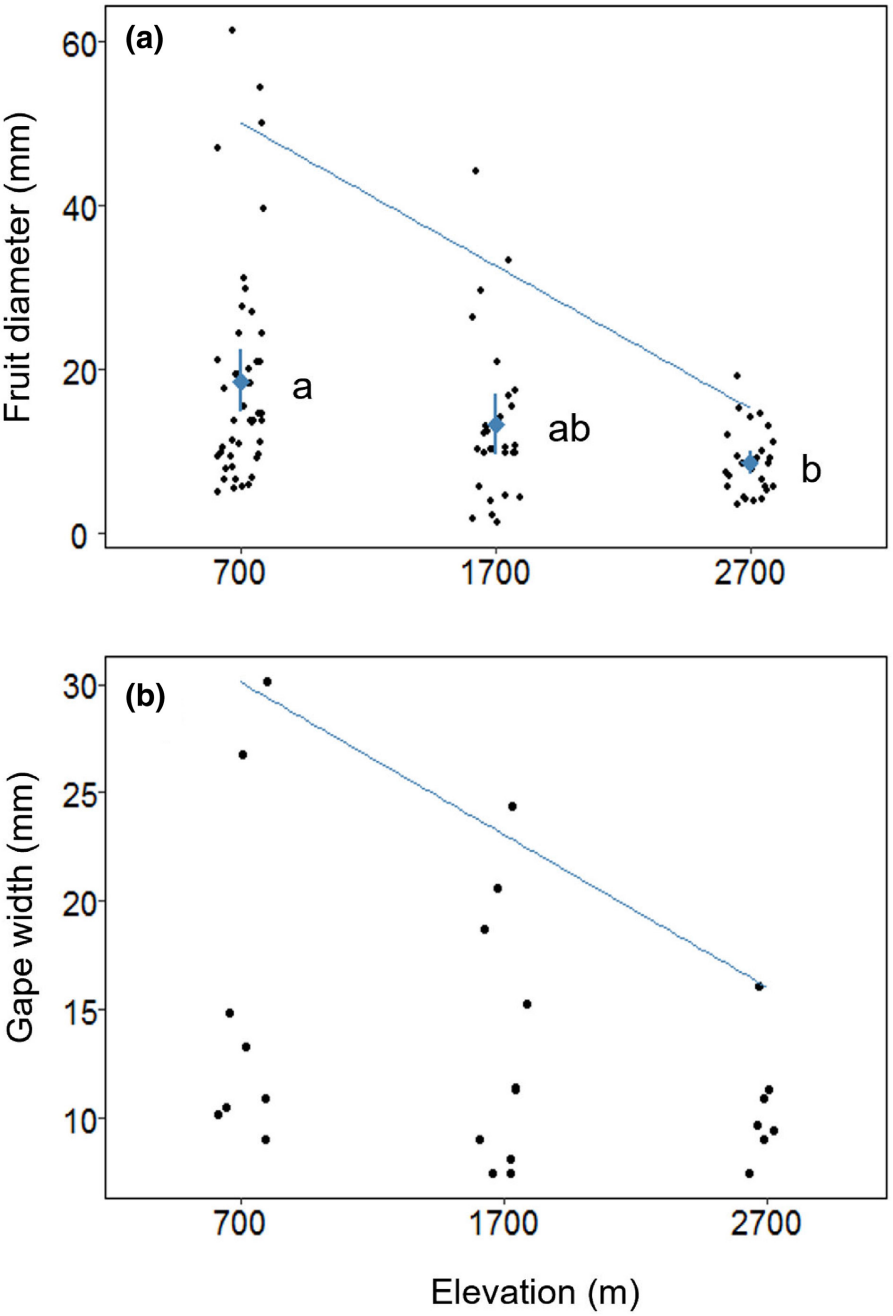
Fruit diameter of fruiting plants (a) and gape widths of understory frugivores (b) at each of the three elevations. (a) Mean diameter of ripe fruits (as measured along a fruit's secondary axis) for a given plant species at each elevation is represented by black circles. The overall elevational mean fruit diameter, weighted evenly per plant species, is denoted by blue diamonds, with error bars representing 95% confidence intervals. The diagonal blue line represents the 95th linear quantile. Letters denote statistically significant differences in overall mean fruit diameter between elevations (*p* < .05), after adjusting using Tukey pairwise comparisons. (b) Black circles here denote the mean adult gape width of each obligate frugivorous bird species recorded at an elevation (*N* = 8 species at 700 m, 10 at 1700 m, and 7 at 2700 m). As with fruit diameter, the diagonal blue line represents the 95th linear quantile. Community‐weighted mean values of fruit diameter and frugivore gape width are displayed in the appendices (Figure A4).

**FIGURE A4:**
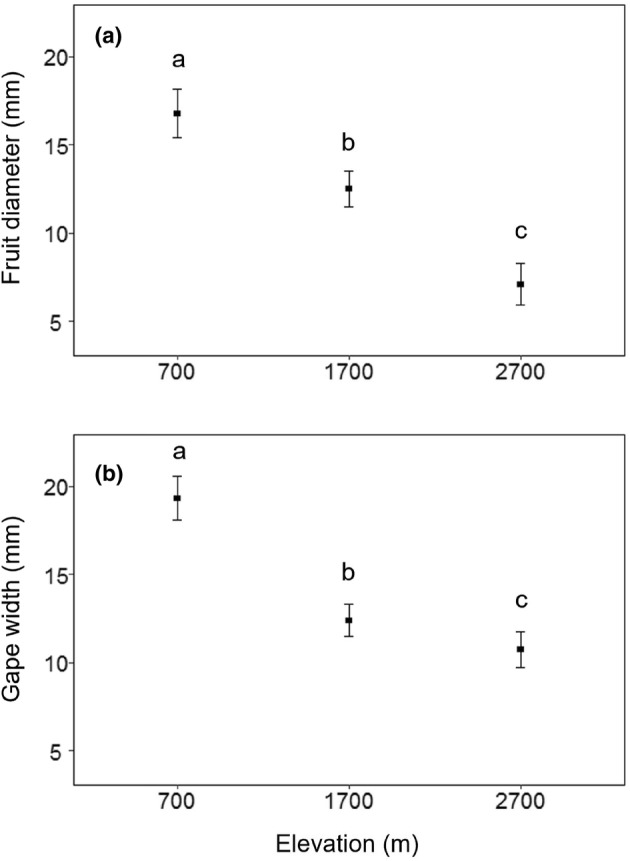
Community‐weighted mean fruit diameter of fruiting plants (a) and understory frugivore gape widths (b) at each of the three elevations. Black squares denote mean values weighted by species' relative abundances. Average values for each individual fruiting plant from which fruit was collected were used to calculate the mean across all individual plants. Error bars represent 95% confidence intervals. Letters above points denote statistically significant differences between elevations (*p* < .05).

The mean diameter of fruits per fruiting plant species decreased steadily and significantly with increasing elevation (GLM_species fruit diameter_, *p* < .01; Figure 3a, Table A1). This decrease appears to be driven primarily by a decrease in maximal rather than minimal fruit diameter (95th percentile, *p* < .01; Figure 3a). Community‐weighted mean fruit diameter also showed a significant decrease with elevation (GLM_community fruit diameter_, *p* < .01; Figure A4a).

#### 3.1.3 Frugivore gape width

Mean gape width of understory frugivore species showed a decreasing, although non‐significant, trend with elevation (GLM_species gape width_, *p* = .27; Table A1). Similar to the patterns observed with fruit size, this decrease appears to be driven mainly by a significant loss of large‐gaped frugivores with increasing elevation (95th percentile, *p* = .04; Figure 3b). Meanwhile, community‐weighted mean gape width decreased significantly with elevation (GLM_community gape width_, *p* < .01; Figure A4b).

#### 3.1.4 Frugivore abundance

**FIGURE 4:**
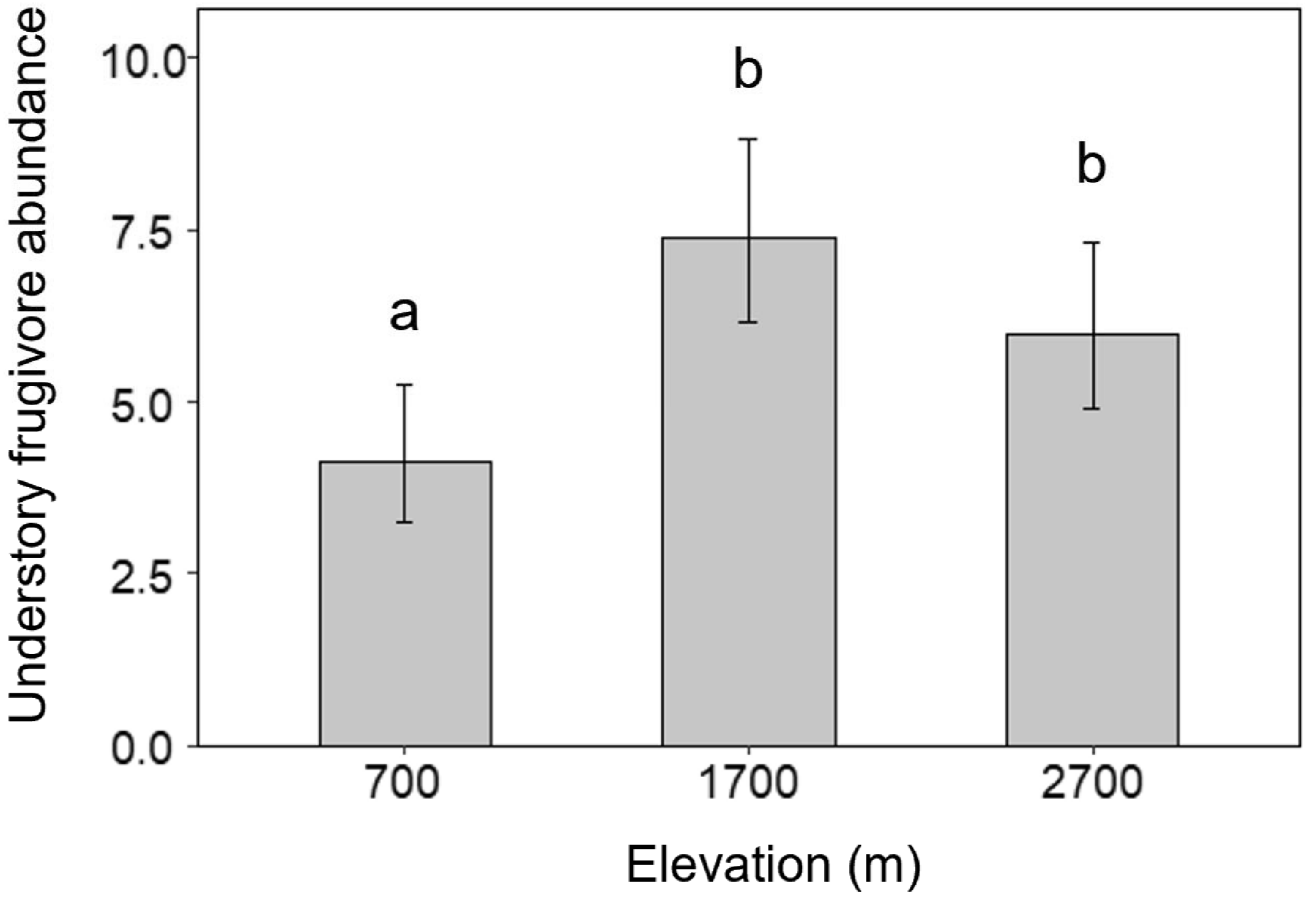
Abundance of frugivorous birds occurring in the understory at each elevation, measured as the mean abundance of obligate understory frugivores recorded (seen or heard) per point count. Error bars represent 95% confidence intervals. Letters above bars denote the significance of multiple comparisons, after adjusting using Tukey pairwise comparisons.

**FIGURE 5:**
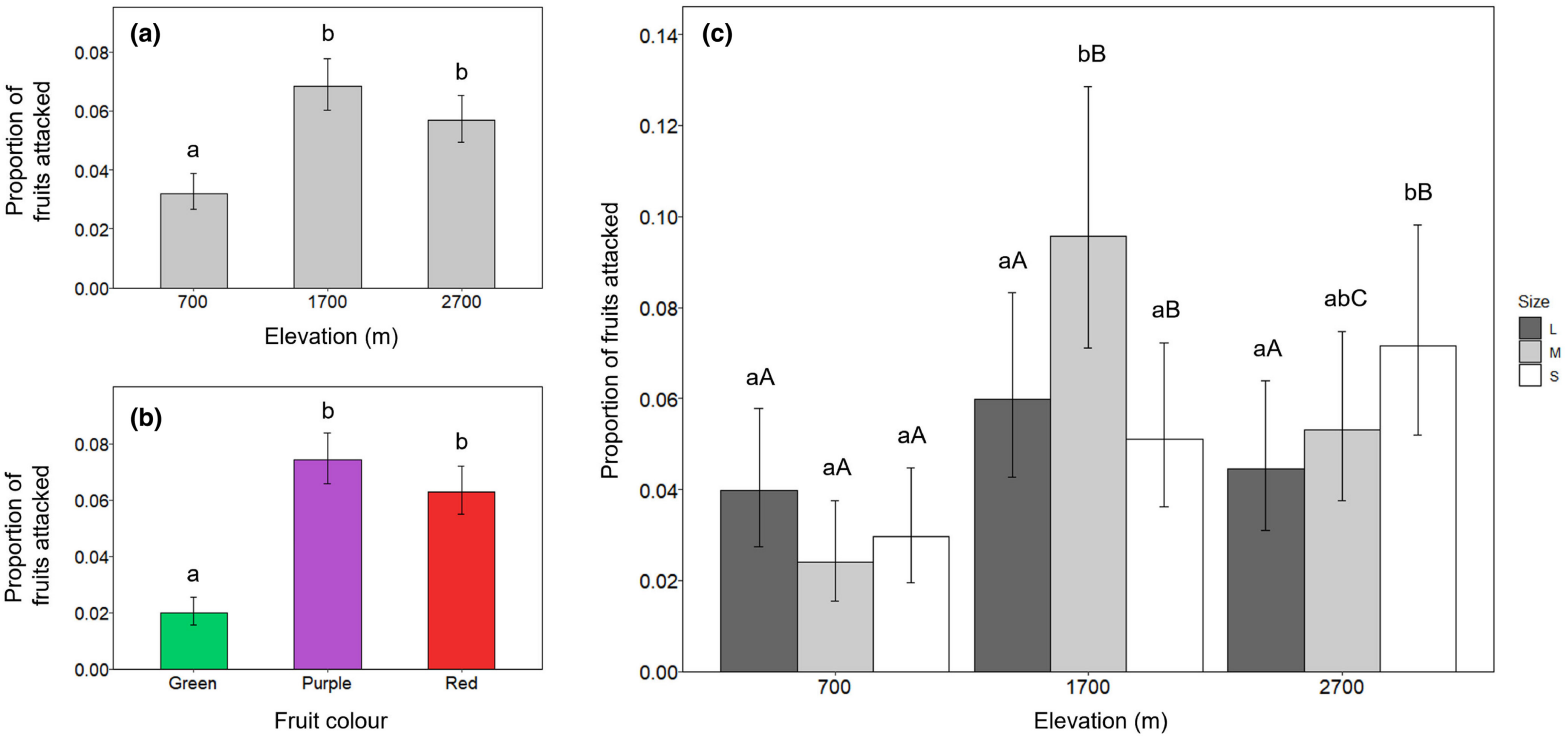
Patterns of attack rates by birds on artificial fruits across elevations (a), fruit colors (b), and fruit sizes at each elevation (c). Mean proportion of fruits showing evidence of bird feeding attempts per 30‐fruit cluster is displayed. Across all elevations combined, fruit size did not significantly affect attack rates, so is not plotted here. Error bars represent 95% confidence intervals in all cases. Letters above bars denote the significance of multiple comparisons between attack rates, after adjusting using Tukey pairwise comparisons. In part (c), dark gray bars represent large fruits, light gray bars represent medium fruits, and white bars represent small fruits; lower‐case letters represent significantly different attack rates on fruits of each size category at a given elevation, and upper‐case letters represent significantly different attack rates between each elevation for a given size category.

Elevation had a significant effect on the abundance of frugivorous understory birds (GLM_frugivore abundance_, *p* < .01; Figure 4, Table A1). Indeed, understory frugivore abundance showed a markedly similar pattern to that of overall attack rates on artificial fruits (Figures 4 and 5a). As with attack rates, frugivore abundance was highest at 1700 m and 2700 m and significantly lower at 700 m.

#### 3.1.5 Artificial fruit exposures

We performed two exposures of 4860 artificial fruits along the whole elevation gradient, during which 510 fruits were attacked by birds and 241 fruits were attacked by other taxa: arboreal mammals (83 fruits), bats (30), and arthropods (128) (Table A2). Eleven fruits were missing entirely and were excluded from analyses.

No collinearity was detected among fruit color, fruit size, and elevation (Table A3). Avian attack rates on artificial fruits—measured as the mean proportion of fruits per 30‐fruit cluster showing evidence of feeding attempts—were significantly lower at 700 m in comparison with attack rates at 1700 and 2700 m, but attack rates at 1700 and 2700 m were similar (GLM_attack rates_; Figure 5a; Table 1). Along the whole gradient, purple and red fruits were attacked more than green fruits, but similarly to each other (Figure 5b, Table 1). Elevation had little to no effect on the relative attack rates by birds on different colored fruits (Table 1). Green fruits were consistently the least attacked (Figure 5b). Purple fruits were significantly more attacked than red fruits only at 1700 m (*p* = .05). There was similarly little interaction between color and size of attacked fruits, although this interaction was near significant (*p* = .06; Table 1). Green fruits were again least attacked across size categories, and attack rates on green fruits did not differ with fruit size. Attack rates on purple and red fruits were similar for all fruit sizes.

**TABLE 1:** Results of generalized linear model for bird attack rates on artificial fruits including fixed effects of fruit size, fruit color, elevation, and their interactions

Parameter	Deviance	*p* value	Multiple comparisons	Estimate	SE	Adjusted *p*‐value
Size	2.56	.28				
Color	123.22	**<.01**	G vs. P	−1.38	0.14	**<.01**
			G vs. R	−1.2	0.15	**<.01**
			P vs. R	0.18	0.1	.16
Elevation	48.28	**<.01**	700 vs. 1700	−0.79	0.13	**<.01**
			700 vs. 2700	−0.61	0.13	**<.01**
			1700 vs. 2700	0.19	0.11	.18
Size:Color	9.25	.06				
Size:Elevation	25.1	**<.01**	700:L vs. 700:M	0.51	0.25	.1
			700:L vs. 700:S	0.3	0.24	.41
			700:M vs. 700:S	−0.21	0.26	.71
			1700:L vs. 1700:M	−0.48	0.17	**.01**
			1700:L vs. 1700:S	0.16	0.19	.67
			1700:M vs. 1700:S	0.64	0.17	**<.01**
			2700:L vs. 2700:M	−0.18	0.2	.64
			2700:L vs. 2700:S	−0.49	0.19	**.03**
			2700:M vs. 2700:S	−0.31	0.18	.21
Color: Elevation	6.71	.15				
Size:Color: Elevation	7.1	.53				

*Note*: We present deviance values for each fixed effect and each pairwise/triple interaction between effects. Estimate and standard error of multiple comparisons are displayed for fixed effects and interactions that were significant at *p* < .05. *P*‐values for multiple comparisons are adjusted using Tukey pairwise comparisons. Significant results are displayed in bold. Results of the generalized linear model for the subset of fruits held in the beak by birds are presented in the appendices (Table A4).

Considering data from along the whole gradient, there was no significant difference between attack rates on different‐sized artificial fruits (*p* = .28). However, elevation in combination with fruit size had a significant effect on the number of attacked fruits (Table 1). Birds showed no preference for fruit size at 700 m and attacked medium‐sized fruits significantly more often than small and large fruits at mid‐elevations (1700 m). Finally, small fruits were attacked significantly more often than large fruits at 2700 m, with medium fruits showing an intermediate attack rate (Figure 5c).

**FIGURE 6:**
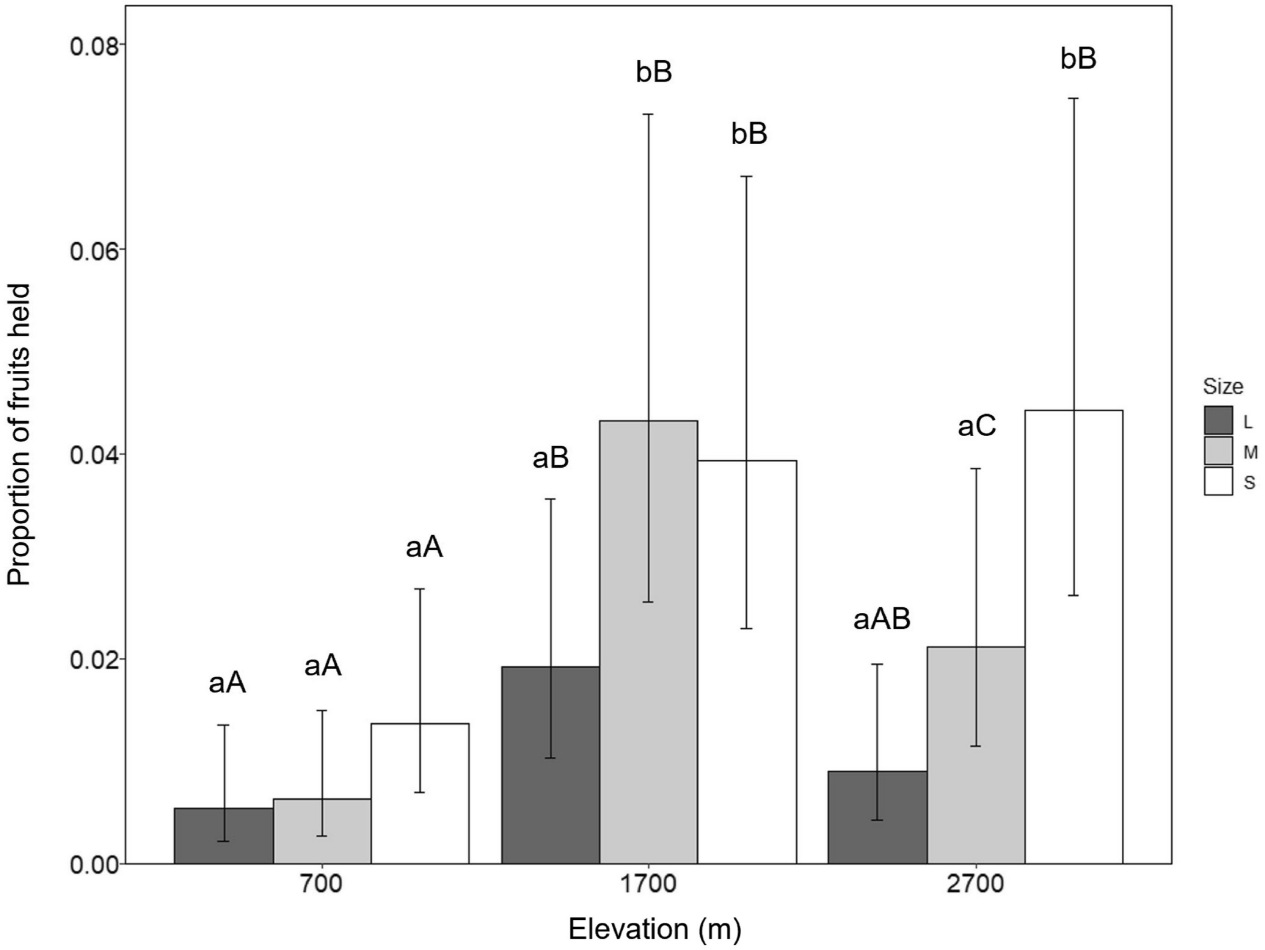
Proportion of differently sized artificial fruits held in the beak by birds at three elevations. Mean proportion of held fruits per 30‐fruit cluster is displayed. Dark gray bars represent large fruits, light gray bars medium fruits, and white bars small fruits. Error bars represent 95% confidence intervals. Letters above bars denote the significance of multiple comparisons between holding rates, after adjusting using Tukey pairwise comparisons. Letters represent significantly different holding rates on fruits of each size category at a given elevation (lower case) and between each elevation for a given size category (upper case).

When restricting attack rates only to fruits that were held in the beak (indicating potential dispersal), we found the interaction between fruit size and elevation to again be the most important interaction (GLM_holding rates_; Figure 6; Table A4), although in this case, it was marginally significant overall (*p* = .05). Multiple comparisons of “held” fruit size within elevations showed a pattern similar to that of overall attack rates, but with some noteworthy differences. At 700 m, holding rates did not differ between fruit sizes. Medium fruits were held significantly more than large fruits at 1700 m, but at a similar rate to small fruits. At 2700 m, small fruits were held significantly more than medium fruits, which were in turn held significantly more than large fruits (Figure 6; Table A4).

## 4 DISCUSSION

The variation in fruit traits (e.g., color and size) across climatic gradients is fairly well known (Chen et al., 2016; Lu et al., 2019), but our understanding of the changes in frugivore preferences remains poor. Our study is the first to report community‐level preferences for fruit traits among frugivorous birds along an elevational gradient and to relate them to the actual traits of fleshy fruits present at different elevations. Birds preferred red and purple fruits to green fruits, and their color preferences did not shift with elevation, despite clear elevational changes in the prevalence of different colored real fruits. However, bird size preferences did change with elevation: birds preferred smaller fruits at higher elevation, intermediate‐sized fruits at mid‐elevation, and showed no size preference at lower elevations. Meanwhile, we found that understory frugivory rates increased with elevation, broadly corresponding to patterns of understory frugivore abundance (Smith & McWilliams, 2014).

### 4.1 Overall attack rates

The overall attack rate on artificial fruits of 5.3% by birds and 2.5% by other taxa (arboreal mammals, bats, and arthropods) after 72 h is comparable to the results of other studies. In large forest fragments of Brazilian Atlantic forests, birds were responsible for attacks on 5% of artificial fruits after 96 h (Galetti et al., 2003). In central Amazonia, birds attacked 10% of fruits in 6 days (Arruda et al., 2008). A higher number of feeding attempts was detected in a continuous Brazilian forest, where birds attacked ca. 11% of fruits after 3 days (Alves‐Costa & Lopes, 2001). As in these studies, we found a significant effect of fruit color and physical environment (elevation in this case) on the number of attacks on fruits.

We recorded lower overall attack rates on fruits at 700 m than at higher elevations. Considering that frugivory increases with increasing frugivore abundance (Smith & McWilliams, 2014), such a trend initially seems to contradict existing data suggesting that frugivorous bird abundance generally decreases with increasing elevation (Sam et al., 2017; Terborgh, 1977). However, the placement of artificial fruits within a few meters of the ground means this study specifically provides a representation of understory frugivory. Data from the same sites surveyed in this study show that abundances of avian obligate frugivores foraging within the understory only (i.e., excluding canopy feeders) actually correlate closely with frugivory rates recorded between elevations. These data suggest that at lower elevations, the forest canopy hosts the majority of frugivory interactions, whereas understory frugivory becomes relatively more important as elevation increases and the canopy becomes more open (Sam et al., 2019).

### 4.2 Color and elevation

Overall, we found that birds attacked red and purple fruits more often than green fruits. Indeed, a preference for red over green fruits has been commonly recorded by studies on avian frugivory (Amico et al., 2011; Arruda et al., 2008; Duan et al., 2014; Janson, 1983; Lomáscolo et al., 2008; McPherson, 1988; Wheelwright & Janson, 1985). However, attack rates on red and purple fruits were not significantly different from one another. Furthermore, elevation did not affect the attack rates on different colored fruits. Additionally, bird color preferences did not correspond to the color of the most common fruits at each elevation. This suggests that birds are exerting little selective pressure on fruit color at a community level and that other factors may be more important than frugivory in determining fruit color.

That our results show little preference between purple and red fruits regardless of elevation and fruit size seems to suggest a lack of innate preferences between these colors at the community level. We also observed universally low attack rates on green fruits, including no preference at 700 m where green fruits are relatively common. However, it should be noted here that the abundance of green fruits in lowlands may reflect a higher abundance of mammalian frugivores (such as bats) at these elevations. Indeed, our data found a higher attack rate by mammals on green fruits at 700 m (2.7%) than any other color/elevation combination (Table A2). Nevertheless, green fruits contrast less with a background of foliage than red or purple fruits, and as primarily visual foragers birds are likely to see red or purple fruits more clearly against such a background (Nevo et al., 2018). Our data are therefore consistent with a stochastic explanation for fruit color choice; i.e., birds randomly selecting fruits that are easily noticeable to them, as has been demonstrated in certain species (Schmidt et al., 2004). However, our findings do not discount the possibility that red and purple fruits are simply characterized by similar nutritional rewards, resulting in a lack of clear preferences by birds. In addition, the visual assessment of colors in this study ignores ultraviolet light which is often reflected by purple fruits (Siitari et al., 1999). Further experiments using a wider range of artificial fruit colors may help to more fully reveal whether stochastic or deterministic factors are responsible for apparent fruit color preferences of birds.

The lack of a correlation between real fruit colors and bird color preferences in this study suggests that other factors may account for the differences in real fruit colors observed between elevations. For example, many plant traits (including fruit traits) are known to show evidence of phylogenetic signal, whereby closely related plants may display similar traits regardless of environmental factors or selection pressures between trophic levels (Blomberg et al., 2003; Jordano, 1995). However, fruit color specifically has been shown by several studies to be evolutionarily labile (Cazetta et al., 2012; Ordano et al., 2017; Stournaras et al., 2013), perhaps due to the high versatility of the biosynthetic pathways for plant pigments (Rausher, 2008). Alternatively, fruit color may reflect an adaptation to abiotic, rather than biotic factors (Burns, 2015; Valenta et al., 2018). Anthocyanins, which are responsible for blue, purple, and red colors in fruit, have been found to vary as a function of decreasing latitude and increasing elevation (Zoratti et al., 2015), suggesting that high light levels favor their production. Such a pattern could explain the prevalence of purple and red fruits at higher elevations in our study sites where the canopy is more open and a greater proportion of the forest receives direct sunlight.

### 4.3 Size and elevation

We found avian community‐level fruit size preferences to mirror a decreasing trend in frugivorous bird gape width, suggesting community‐scale trait matching. We also found an association between fruit size preferences and the diameter of actual fruits along the elevational gradient, suggesting a potential selective effect of frugivores' fruit size preferences on fruit size. The results must be interpreted with some caution, as the trend for larger understory frugivore gapes (and to an extent the trend for larger fruits) recorded at lower elevations is driven by relatively few species. Therefore, these patterns could simply represent an accident of biogeography, not necessarily common to other elevational gradients. In addition, the results of this study do not discount the alternative possibility that fruit size is constrained by factors other than frugivore dispersal, and that observed changes in frugivore gape width and feeding preferences are in response to the available fruits at each elevation. Nevertheless, to our knowledge, this study constitutes the first experimental evidence of bird preferences for fruit size on a community scale.

Trait matching should predict sites with large‐gaped birds to show higher attack rates on large fruits and vice versa (Dehling, Töpfer, et al., 2014). While this trend was apparent at higher elevations, we found only weak evidence of frugivores feeding preferentially on large fruits at 700 m. This is despite the diameter of the large artificial fruits being based on the mean diameter for real fruits measured at that elevation. There are a few possible explanations for this. Firstly, some of the largest fruits recorded at 700 m are likely to be mammal dispersed rather than bird dispersed, meaning the mean diameter of bird‐dispersed fruits could actually be lower than was measured. Secondly, our data show that while maximal frugivore gape width indeed decreases with increasing elevation, small‐gaped frugivores are still present at low elevations. Thus, a lack of a clear community preference for large fruits at 700 m is consistent with the community displaying a wide range of gape sizes, even if large birds individually tend to preferentially select larger fruits (Burns, 2013). It is also important to note that large‐bodied (and thus large‐gaped) frugivores are likely to be less abundant than smaller species (White et al., 2007). This means community‐level results could be skewed toward small fruits and small‐gaped frugivores. As shown in Figure 3b, at 700 m, only two species of understory frugivore were recorded with a gape width larger than 19 mm (the diameter of “large” fruits in our study). If characterized by low species abundances, these species should have only a limited effect on frugivory rates overall.

If we are to consider frugivory mutualism from the perspective of plants, the feeding behavior employed by frugivores is important (Rey & Gutierrez, 1996). A bird that swallows a fruit whole is far more likely to fulfill a seed dispersal function than one that pecks it (Simmons et al., 2018). Dispersal of seeds results in lower‐density‐dependent mortality of seedlings and thus is an important component of plant fitness (Beckman & Rogers, 2013; Howe & Smallwood, 1982). Therefore, fruit swallowing is likely to act as a positive selective pressure on fruit traits, whereas pecking is not. When considering a subset of artificial fruits that were held in the beak and thus potentially able to be dispersed, frugivore gape range and fruit size preferences show a strikingly similar pattern across elevations. As maximal frugivore gape width decreases with increasing elevation, so too does the maximal size of fruits held in the beak, while the minimal gape width and minimal held fruit size (small) do not change. Our results, therefore, suggest that “community gape limitation” may be a factor limiting maximal fruit size. In terms of selective pressure, an upper size limit of fruits is more important than a lower size limit in determining a plant's chance of dispersal success. This mirrors the pattern of individual gape limitation, whereby large‐gaped frugivores are able to disperse small fruits but not vice versa (Wheelwright, 1985).

While focusing on the relationship between fruit size and gape width, our results do not preclude the notion that fruit size may be constrained by factors other than frugivory. For example, tropical mountains such as Mt Wilhelm are characterized by a rapid turnover of abiotic conditions such as temperature and precipitation with changing elevation. It is known within certain plant species and genera that fruit size may vary according to factors such as water availability (Larrinaga & Guitián, 2016) and fire exposure (Murray & Gill, 2008). As with fruit color, light availability may also be relevant—large fruits allow the production of large seeds, which are associated with enhanced seedling survivorship at low light intensities such as those found on the forest floor (Foster, 1986). Natural enemies could also play a role in selecting seed (and thus fruit) size. Large seeds are more tolerant to predation by rodents and beetles (Harms & Dalling, 1997; Mack, 1998), and produce seedlings with greater vigor (Lopes Souza & Fagundes, 2014; Pizo et al., 2006). However, large fruits have in some cases been shown to be more vulnerable to attack by fungal pathogens (Beckman & Muller‐Landau, 2011).

The patterns described here highlight a trade‐off faced by fleshy‐fruited plants in tropical forests that can be broadly considered in terms of “quality” versus “quantity.” The developmental and protective benefits afforded by having large fruits and seeds may be offset by the fact that small fruits can be produced in greater numbers for the same energy cost. Additionally, as our results demonstrate, having large fruits limits potential avian dispersal agents to only a subset of the bird community, whereas having smaller fruits does not (Muñoz et al., 2017; Wheelwright, 1985). While large frugivores, typically having large range sizes, may provide “high quality” long‐distance dispersal (Wotton & Kelly, 2012), limiting potential dispersers in this way represents a risky strategy for plants, especially at sites with naturally low abundances of large frugivores (such as high elevations).

The bi‐directional nature of the frugivory mutualism means that while birds have the potential to apply selective pressure on fruit size, the opposite may also be true: fruit size could conceivably constrain the range of frugivores present at an elevation according to their gape width. While it is difficult to untangle cause and effect at the community scale in the absence of detailed knowledge of the functional evolutionary histories of the birds and plants in the study area, it is likely that both processes occur to some extent. However, the asymmetrical nature of gape limitation means that birds are likely to inherently pose a stronger selection pressure on fruit size than the other way around. Larger‐gaped birds may still survive by consuming fruits smaller than their gape width, while larger fruits cannot be dispersed if there are no dispersers with gapes large enough to swallow them (Simmons et al., 2018).

### 4.4 A note on artificial fruits

The use of artificial fruits is a useful tool for ascertaining feeding preferences of frugivorous birds at the community level without resorting to invasive techniques which may affect birds' behavior. However, there are a few limitations to the approach. Unlike lab‐based studies, community‐based approaches such as this do not identify individual feeding interactions, which would enable more direct functional comparisons between fruits and their dispersers. Additionally, our study, in common with others using similar methodologies, is limited by the placement of artificial fruits relatively close to the ground. This neglects information on the feeding preferences of canopy‐feeding frugivores, which form a very important component of avian frugivory, especially in lowland rainforests (Schleuning et al., 2011; Shanahan & Compton, 2001). This also highlights a weakness of our study design, whereby data on actual fruits included those collected from all layers of the forest, meaning the data are not directly comparable to the understory frugivory represented by the attack rates on artificial fruits. An extension of our experimental methodology to encompass canopy as well as understory frugivory, while logistically difficult, would doubtless provide a more complete picture of avian community‐level fruit preferences.

## 5 CONCLUSIONS

This study represents the first attempt to record community‐wide preferences of frugivorous birds for fruit traits along an elevational gradient. We have shown that at the community scale, birds do not preferentially select artificial fruits corresponding to the color of prevalent real fruits. This lack of correspondence suggests that while birds are able to adapt their feeding behavior based on the fruits locally available, inherent avian color preferences are probably broad and inflexible and thus unlikely to be able to select specific fruit colors. In contrast, we showed fruit size preferences of birds do correspond to real fruit size along the gradient, and that preferences are consistent with gape limitation hypothesis. A result is that smaller and smaller fruits are preferred with increasing elevation. Furthermore, this pattern extends to fruits held in the beak by birds and thus able to be dispersed, suggesting the potential for birds to act as a selective pressure on fruit size. This result demonstrates a trade‐off for plants between maximizing seed size and maintaining the likelihood of dispersal, especially at high elevations. Nevertheless, further experimental study is needed if we are to untangle explicitly whether a lack of large fruits at high elevations is a result of selection by birds or due to other factors such as environmental constraints.

## AUTHOR CONTRIBUTIONS

**Richard J Hazell:** Conceptualization (equal); data curation (equal); formal analysis (equal); investigation (equal); methodology (equal); project administration (equal); visualization (equal); writing – original draft (equal); writing – review and editing (equal). **Katerina Sam:** Conceptualization (equal); funding acquisition (equal); methodology (equal); project administration (equal); supervision (equal); visualization (equal); writing – review and editing (equal). **Rachakonda Sreekar:** Data curation (equal); formal analysis (equal); visualization (equal); writing – review and editing (equal). **Samson Yama:** Investigation (equal); methodology (equal); writing – review and editing (equal). **Wulan Koagouw:** Project administration (equal); supervision (equal); visualization (equal); writing – review and editing (equal). **Alan Stewart:** Funding acquisition (equal); project administration (equal); supervision (equal); writing – review and editing (equal). **Mika Peck:** Funding acquisition (equal); methodology (equal); project administration (equal); supervision (equal); writing – review and editing (equal).

## CONFLICT OF INTEREST

The corresponding author confirms on behalf of all authors that there have been no involvements that might raise the question of bias in the work reported or in the conclusions, implications, or opinions stated.

## Data Availability

All data and scripts supporting the findings of this study are openly available in the FigShare digital repository at the following DOI: https://doi.org/10.6084/m9.figshare.c.6410138. This collection includes: Sample locations and abundance and trait data of real fruits and birds; Experimental artificial fruit exposure data (attack rates); R code for calculation of statistical analyses and creation of plots, plus associated files.

## APPENDIX A

**TABLE A1:** Results of generalized linear models (GLMs) for obligate understory frugivore abundance and gape width, and real fruit diameter and relative abundances of real green, purple, and red fruits at the three study sites

Parameter	Deviance	*p* value	Multiple comparisons	Estimate	SE	Adjusted *p*‐value
Frugivore Abundance	15.01	**<.01**	700 vs. 1700	−0.58	0.15	**<.01**
			700 vs. 2700	−0.37	0.16	**.05**
			1700 vs. 2700	0.21	0.14	.29
Frugivore Gape Width (species weighted)	98.63	.27				
Frugivore Gape Width (community weighted)	3128.8	**<.01**	700 vs. 1700	6.95	0.78	**<.01**
			700 vs. 2700	8.6	0.81	**<.01**
			1700 vs. 2700	1.66	0.7	**.05**
Fruit Diameter (species weighted)	1741.4	**<.01**	700 vs. 1700	5.35	2.55	.09
			700 vs. 2700	9.95	2.58	**<.01**
			1700 vs. 2700	4.61	2.87	.24
Fruit Diameter (community weighted)	6530.1	**<.01**	700 vs. 1700	4.29	0.88	**<.01**
			700 vs. 2700	9.67	0.93	**<.01**
			1700 vs. 2700	5.39	0.8	**<.01**
Fruit Color: Green	15.35	**<.01**	700 vs. 1700	1.36	0.34	**<.01**
			700 vs. 2700	1.32	0.34	**<.01**
			1700 vs. 2700	−0.04	0.4	.99
Fruit Color: Purple	157.11	**<.01**	700 vs. 1700	2.9	0.81	**<.01**
			700 vs. 2700	−2.21	0.45	**<.01**
			1700 vs. 2700	−5.11	0.74	**<.01**
Fruit Color: Red	1.32	.52				

*Note*: Elevation was the single fixed effect included in each GLM, for which deviance values are displayed here. Estimate and standard error of multiple comparisons between elevations are displayed when the effect of elevation was significant at *p* ≤ .05. *P*‐values for multiple comparisons are adjusted using Tukey pairwise comparisons. Significant results are displayed in bold.

**TABLE A2:** Attack rates on artificial modeling clay fruits divided by frugivore type and elevation

	Category	Birds	Arboreal mammals	Bats	Arthropods	All taxa
**700 m**	LG	9	15	3	7	34
	MG	5	2	7	9	23
	SG	5	0	1	4	10
	LP	17	0	3	3	23
	MP	10	5	7	14	36
	SP	12	2	2	10	26
	LR	18	1	3	6	28
	MR	12	2	1	8	23
	SR	16	2	0	9	27
	**700 m Total**	**104**	**29**	**27**	**70**	**230**
**1700 m**	LG	6	3	0	1	10
	MG	10	1	0	0	11
	SG	8	1	0	3	12
	LP	25	1	0	1	27
	MP	59	1	1	17	78
	SP	31	1	0	8	40
	LR	34	17	0	0	51
	MR	32	2	0	1	35
	SR	17	0	0	6	23
	**1700 m Total**	**222**	**27**	**1**	**37**	**287**
**2700 m**	LG	6	1	0	0	7
	MG	5	10	0	0	15
	SG	11	0	0	3	14
	LP	19	4	0	6	29
	MP	28	1	0	1	30
	SP	40	1	0	0	41
	LR	24	5	2	7	38
	MR	25	3	0	3	31
	SR	26	2	0	1	29
	**2700 m Total**	**184**	**27**	**2**	**21**	**234**
**All Elevations Total**		**510**	**83**	**30**	**128**	**751**

*Note*: “Category” describes the fruit color/size combination: L = large, M = medium, S = small; G = green, P = purple, and R = red. Numbers in columns represent the number of artificial fruits showing evidence of a feeding attempt by members of the taxon in question, of a total of 360 (180*2 exposures) for each elevation/color/size combination. For example, at 700 m, 9 of 360 exposed large green fruits showed evidence of attempted frugivory by birds. Numbers in bold represent total attack rates per taxon at each elevation, and overall.

**TABLE A3:** Results of collinearity test for the variables of fruit size, fruit color, and elevation used in the model GLM_attack rates_

	GVIF	df	GVIF^(1/(2*df))^	(GVIF^(1/(2*df))^)^2^
Fruit size	5.887	2	1.558	2.426
Fruit color	1	2	1	1
Elevation	9.277	2	1.745	3.046

*Note*: We present generalized variance inflation factors (GVIF) for each variable, together with their scaled values (GVIF^(1/(2*df))^), where df is the degrees of freedom associated with the variable. The squared scaled values in the final column are equivalent to standard VIF values, where numbers above 5 indicate mild collinearity with at least one other variable and numbers above 10 indicate severe collinearity.

**TABLE A4:** Results of generalized linear model (GLM) for the subset of bird attack rates in which artificial fruits were held in the beak, including fixed effects of fruit size, fruit color, elevation, and their interactions

Parameter	Deviance	*p* value	Multiple comparisons	Estimate	SE	Adjusted *p*‐value
Size	34.15	**<.01**	L vs. M	−0.61	0.24	**.03**
			L vs. S	−1.08	0.22	**<.01**
			M vs. S	−0.47	0.19	**.03**
Color	91.07	**<.01**	G vs. P	−1.98	0.25	**<.01**
			G vs. R	−1.45	0.26	**<.01**
			P vs. R	0.53	0.15	**.00**
Elevation	54.04	**<.01**	700 vs. 1700	−1.42	0.23	**<.01**
			700 vs. 2700	−0.97	0.25	**<.01**
			1700 vs. 2700	0.45	0.17	**.02**
Size:Color	9.25	.06				
Size:Elevation	9.43	**.05**	700:L vs. 700:M	−0.16	0.56	.96
			700:L vs. 700:S	−0.93	0.49	.14
			700:M vs. 700:S	−0.77	0.46	.21
			1700:L vs. 1700:M	−0.82	0.27	**<.01**
			1700:L vs. 1700:S	−0.72	0.27	**.02**
			1700:M vs. 1700:S	0.1	0.22	.90
			2700:L vs. 2700:M	−0.85	0.38	.07
			2700:L vs. 2700:S	−1.6	0.35	**<.01**
			2700:M vs. 2700:S	−0.75	0.26	**.01**
Color:Elevation	6.59	.16				
Size:Color:Elevation	14.23	.08				

*Note*: We present deviance values for each fixed effect and each pairwise/triple interaction between effects. Estimate and standard error of multiple comparisons are displayed for fixed effects and interactions that were significant at *p* ≤ .05. *P*‐values for multiple comparisons are adjusted using Tukey pairwise comparisons. Significant results are displayed in bold.
